# Overweight/Obesity and associated factors among preschool children in Gondar City, Northwest Ethiopia: A cross-sectional study

**DOI:** 10.1371/journal.pone.0182511

**Published:** 2017-08-07

**Authors:** Muluken Bekele Sorrie, Melkie Edris Yesuf, Tsgehana GebreGyorgis GebreMichael

**Affiliations:** 1 Arbaminch University College of Medicine and Health Sciences Department of Midwifery, Arbaminch, Ethiopia; 2 University of Gondar College of Medicine and Health Sciences Institute of Public Health, Gondar, Ethiopia; International Nutrition Inc, UNITED STATES

## Abstract

**Background:**

Overweight and obesity among children has emerged as one of the most serious public health concerns in the 21st century, which is a predictor of adulthood obesity, morbidity and mortality. The objective of this study was to assess the prevalence of overweight /obesity and associated factors among preschool children.

**Methods:**

A community based cross-sectional study was conducted in Gondar City from February 14 to March 4, 2016. Multi stage sampling technique was used to select a total of 504 preschool children. Data were collected using structured interviewer administered questionnaire and anthropometric measurements. Data were entered using Epidata version 3.1 and analyzed using SPSS version 20 and WHO 2007 Anthro version 2.0.4 software. Both bivariate and multivariate logistic regression analysis were performed to identify associated factors. P values <0.05 with 95% confidence level were used to declare statistical significance.

**Results:**

A total of 500 study participants were included with 99.2% response rate and 51.6% were girls while 48.4% were boys. The mean (±SD) age of participants was 47.68 ±7.19 months. The combined prevalence of overweight /obesity was 13.8% (95%CI; 10.6, 17.2) the specific being 9.6% for overweight and 4.2% for obesity. The multivariable analysis indicated that the age group between 36–47 months [AOR = 2.38 (95%CI; 1.27,4.46)],high dietary diversity[AOR = 3.73(95%CI;1.15,12.54),consumption of sweet food[AOR = 2.69 (95%CI,1.21, 5.98)],time spent in watching television>2hr/day [AOR = 4.01 (95%CI;2.22, 7.26)] and mother’s education at secondary level [AOR = 0.35 (95% CI; 0.12, 0.96)] were associated with overweight/obesity among preschool children.

**Conclusions:**

Once considered a high income country problem, result of this study in urban city like Gondar reveals that overweight/obesity is on the rise in urban Ethiopia, which indicates the need for formulating preventive programs and policies during a child’s early years.

## Background

Overweight and obesity are defined as abnormal or excessive accumulation of fat which may impair health [1].

Worldwide the prevalence of childhood overweight and obesity increased from 4.2% in 1990 to 6.7% in 2010 and expected to reach 9.1% in 2020 by affecting more than 1.5 billion adults and accounting for 0.7% to 2.8% of healthcare expenditures. The estimated prevalence of at risk of overweight was 21.4% in developed and 13.6% in developing countries in 2010. However the relative percentage change is higher in developing countries which is 8.5% in 2010 and expected to reach 12.7% in 2020, similarly in Asia the prevalence increased to 4.9%from 3.2%. Obesity in preschool children is a growing problem and it is receiving increasing attention which is a predictor of adulthood obesity, morbidity and mortality [2, 3].

Infectious diseases and under-nutrition were still major public health concerns in the developing world. With modernization and industrialization physical activities including occupational, commuting and leisure-time physical activities are reduced substantially, which in turn can have effect on individual life style [4, 5]. In Sub-Saharan countries the prevalence of overweight and obesity is substantially high, even in some countries over nutrition is more common than under nutrition, which shows that nutrition transition is occurring and overweight and obesity are becoming a growing problem in the region [6].

Many factors can be associated with overweight and obesity in preschool children. From this, factors which are on the maternal side were socioeconomic status, level of education, marital status and, maternal smoking during pregnancy. Sex of the child, birth weight and the child's birth rank, area of residence, BMI of parents and some nutritional factors have been also found as factors of childhood overweight and obesity [7, 8].

In Ethiopia, particularly in the study area, there was no information regarding the prevalence of overweight /obesity and its associated factors among preschool children though preschool years are a crucial time when eating and physical activity habits are becoming established.

Therefore this study will help to determine the prevalence and the most important associated factors which have impact on overweight /obesity among preschool children in the study area.

## Methods

Community based quantitative cross sectional study was conducted from February 14 to March 4, 2016 at Gondar City which is located at a distance of 737km from Addis Ababa (capital of Ethiopia). The city were divided in to 12 urban sub cities and 11 rural kebele, in the urban sub cities there were 45,147 under 5 children of this 19,702 were preschool children aged 3–5 years. There were one governmental hospital, one private hospital,14 health centers and 8 health posts in the city during the study period [9].

### Study population

All preschool children who were living in Gondar city were target for the study. All preschool children who were living in selected sub-cities during the data collection period were included in the study. Preschool children who were seriously ill during the study period were excluded from the study.

### Sample size and sampling

Sample size was determined by taking the prevalence of overweight/obesity in a study done on Hawassa City which was 10.7% [10], with 4% marginal error, design effect of 2, 95% CI and a none response rate of 10%. Based on this assumption, the final sample size was 504. Multistage sampling was employed to select preschool children. From the total of 12 sub-cities, four sub-cities selected randomly by using lottery method then sample were allocated to each selected sub city proportionally based on their number of preschool children.

Finally children in every 12 household was enrolled by systematic sampling method and for those households who have more than one preschool children lottery method was used to select one individual for the study, whereas the next household was considered when there was no eligible child in the systematically selected household.

### Data collection tools and procedures

The questionnaire which was administered in the local language included questions that assessed socio-economic and demographic factors, practices of breastfeeding and infant formula feeding of the child, food frequency questionnaire in past one week and children’s dietary diversity which was collected by asking mothers/care givers face to face.

Determination of dietary diversity score (DDS) of the child was started by asking the mother to list all food consumed by the child in the previous 24 hr preceding the survey. Then reported food items were classified into seven food groups as (1) grains, roots and tubers, (2) vitamin A-rich fruits and vegetables, (3) other fruits and vegetables, (4) meat, poultry and fish, (5) eggs, (6) pulses, legumes and nuts, (7) milk and milk products. The score was categorized as Low (DDS <3 food groups consumed), medium (DDS = 3–5 food groups consumed) and high (DDS ≥ 6 food groups consumed).

The global physical activity questionnaire (GPAQ) was used to assess the physical activity pattern among children 3–5 years old through face-to-face interview of mothers/caregivers in the study area. The GPAQ was developed by WHO for physical activity surveillance in developing countries like Ethiopia. Among the questions, we excluded the vigorous sport activity and work related activity part since the study subjects were children 3 to 5 years old.

### Anthropometric measurements

The measurements of height and weight were taken from each child using standardized and calibrated equipment.

Height of children was measured with barefoot by undoing their hair, removing any pins and braids from the hair that could affect the measurement by positioning the subject at the Frankfurt plane using a stadiometer seca (Germany) and recorded to the nearest 0.1cm. Weight of children was measured with light clothing (underwear, t-shirt only) and recorded to nearest 0.1 kg using UNICEF seca digital weighing scale (Germany)[11].

The z-score values for BMI-for-age (BAZ) of children from birth to 60 completed months were generated with WHO child growth standards using WHO 2007 Anthro version 2.0.4 software[12].

The data were collected by 4 diploma nurses one in each sub city and supervised by 2 BSc degree nurses as well as by the principal investigator.

### Data quality management

Data collectors and supervisors were trained for two days by doing standardization exercise in order to minimize errors.

The questionnaire was developed in English and then translated in to Amharic and back to English then review was made for consistency of translation of the language. The data were collected after pretest has been conducted on 26 (5%) of preschool children’s from sub city other than those included.

The principal investigator and supervisors made day to day onsite supervision during the whole period of data collection. The collected data were reviewed and checked for completeness, accuracy and consistency by supervisors and investigator, and weight scale was calibrated and placed in level surface before measurement was performed. Continuous checkup of scales was carried out.

### Data management and analysis

The data were coded on prearranged coding sheet by the principal investigator then entered to Epidata 3.1 and cleaned, processed and analyzed using SPSS version 20. The information obtained was described by using mean, frequencies, proportions, and tables. Body mass index for age was computed by using WHO 2007 Anthro software version 2.0.4 and the WHO standard was used as a reference for classifying the nutritional status of children. Household’s wealth index was determined using Principal Component Analysis (PCA) by considering the household assets, such as quantity of cereal products, type of house, livestock and agricultural land ownership and ownership of various durable goods (radio/tape, television, car, refrigerator, sofa, bicycle, motorcycle, mobile/telephone and others). First, variables were coded between 0 and 1. Then variables entered and analyzed using PCA, and those variables having a communality value of greater than 0.5 were used to produce factor scores. Finally, the factor scores were summed and ranked into tertiles as low, medium and high socio economic status. Bivariate logistic regression analysis was done and variables with P-value of less than 0.2 were included in the multivariate logistic regression analysis to isolate an independent effect of the predictors that showed significant association with overweight/obesity. Backward step-wise method was used and Hosmer and lemeshow goodness of model fit test was 0.85. Finally the odd ratios with 95% confidence intervals were reported to indicate the strength of associations.

## Results

### Socio-demographic and economic characteristics of the study participants

A total of 504 preschool children-pairs with mothers/caregivers (with a response rate of 99.2%) participated in the study. The majority of mothers/caregivers were orthodox Christian (88.8%) followed by Muslims (10.6%). Less than 35% of mothers/caregivers were completed secondary school. The mean (±SD) age of the respondents was 47.7 ± 7.2 SD month ranging from 36 to 60 months and 258 (51.6%) were females and 242 (48.4%) were males (Table 1).

**Table 1:** Socio-economic and demographic characteristics of mothers / care- givers and their children in Gondar City in 2016.

Variable	Frequency	Percent (%)
**Sex of child**		
Female	258	51.6
Male	242	48.4
**Age in month**		
36–47	250	50.0
48–60	250	50.0
**Religion**		
Orthodox	444	88.8
Muslim	53	10.6
Protestant	3	0.6
**Educational status of children**		
Not start school	323	64.6
KG one	155	31.0
KG two	22	4.4
**Educational status of mother**		
No education	89	17.8
Primary education	140	28.0
Secondary education	172	34.4
More than secondary	99	19.8
**Educational status of father**		
No education	54	10.8
Primary education	160	32.0
Secondary education	179	35.8
More than secondary	107	21.4
**Father occupation**		
Private	256	51.2
Merchant	136	27.2
Government employee	93	18.6
Other	15	3.0
**Mother occupation**		
House wife	270	54.0
Private	93	18.6
Government employee	68	13.6
Merchant	64	12.8
Other	5	1.0
**Socio economic status**		
Low	165	33.0
Medium	168	33.6
High	167	33.4
**Family size**		
<5	416	83.2
≥5	84	16.8

Number of total participants, n = 500

### Dietary diversity score and consumption of sweet food by preschool children

The mean dietary diversity score was 4.4 with (±1.4 SD). Vast majority of preschool children consumed grain, root and tuber products (98.2%) and least consumed food was milk and milk products (30.6%). Three hundred forty two (68.4%) of preschool children’s consumed sweet foods in the last 24 hr preceding the survey while 158 (31.6%) did not consume (Table 2).

**Table 2:** Dietary diversity of different food groups for preschool children in the last 24 hrs preceding the survey in Gondar City in 2016.

Food groups	Frequency	Percent (%)
**Foods made from grains, roots and tubers**	491	98.2
**Food made from pulses and nuts**	365	73.0
**Fruits and vegetables**	345	69.0
**Meat, meat products and fish**	335	67.0
**Vitamin A rich fruits and vegetables**	324	64.8
**Egg**	191	38.2
**Milk and milk products**	153	30.6
**Sweet foods**	342	68.4

Number of total participants, n = 500

### Food consumption pattern by preschool children

Majority, 486 (97.2%) of preschool children consumed foods made from cereals every day. More than half of study participants do not consume milk in the past 1 week (Table 3).

**Table 3:** Food consumption pattern of preschool children in the past one week in Gondar City in 2016.

Food groups	Frequency				
	Not taken (No. (%)	Every day (No. (%)	Once a week (No. (%)	Twice a week (No. (%)	3–6 times a week (No. (%)
**Cereals, grains and tubers**	0	486(97.2)	4(0.8)	2(0.4)	8(1.6)
**Meat**	52(10.4)	15(3)	165(33)	164(32.8)	104(20.8)
**Fruits**	46(9.2)	38(7.6)	102(20.4)	174(34.8)	140(28)
**Vegetables**	62(12.4)	7(1.4)	91(18.2)	230(46)	110(22)
**Egg**	198(39.6)	12(2.4)	162(32.4)	93(18.6)	35(7)
**Milk**	288(57.6)	71(14.2)	91(18.2)	34(6.8)	16(3.2)
**Soft drink**	179(35.8)	26(5.2)	162(32.4)	100(20)	33(6.6)
**Sweet foods**	59(11.8)	159(31.8)	77(15.4)	101(20.2)	104(20.8)
**Tea and sugar**	23(4.6)	327(65.4)	22(4.4)	49(9.8)	79)15.8)

Number of total participants, n = 500

### Feeding practices of preschool children

Majority of study participants (97%) were breastfed while 3% of them were not breastfed, among those who breastfed 74.4% were exclusively breastfed until 6 month. 89.6% of study participants were started complementary feeding after 6 month (Table 4).

**Table 4:** Feeding practices of preschool children in Gondar City in 2016.

Variables	Frequency	Percent (%)
**Breast feeding**		
Yes	485	97.0
No	15	3.0
**Duration of exclusive breast feeding**		
The first 3 month	16	3.3
4–6 month	108	22.3
Until 6 month	361	74.4
**Age of starting complementary feeding**		
The first 3 month	21	4.2
4–6 month	31	6.2
After 6 month	448	89.6
**Duration of continued breast feeding**		
<12 month	38	7.8
12–18 month	98	20.2
19–244 month	349	75.0
**Infant formula feeding**		
Yes	123	24.6
No	377	75.4
**Age started infant formula**		
≤3 month	21	17.0
4–6 month	11	9.0
≥6 month	91	74.0
**Duration of infant formula feeding**		
≤3 month	16	13.0
4–11 month	40	32.5
≥12 month	67	54.5

Number of total participants, n = 500

### Physical activity and sedentary behavior

From the total respondents 419 (83.8%) of them used walking to travel from place to place at least for 20 minutes. 31.8% preschool children spent greater than 2 hr/day by watching television (Table 5).

**Table 5:** Physical activity and sedentary behavior of preschool children in Gondar City in 2016.

Variables	Frequency	Percent (%)
**Physical activity related to moving from place to place at least for 20 minute a day**		
Yes	419	83.8
No	81	16.2
**No. of days in a week walking at least for 20 minutes**		
1–2 day	169	33.8
3–4 day	133	26.6
5–7 day	198	39.6
**Minutes in a day for walking**		
≤30 minute	443	88.6
>30 minute	57	11.4
**Moderate sport in a week**		
1–2 day	164	32.8
3–4 day	119	23.8
5–7 day	217	43.4
**Moderate sports in minutes per day**		
≤10 minutes	119	23.8
>10 minutes	381	76.2
**Time spent by watching television in hour per day**		
≤2hr	341	68.2
>2hr	159	31.8

Number of total participants, n = 500

### Prevalence of overweight /obesity

The prevalence of overweight /obesity was 13.8% (95% CI (10.6, 17.2). The age and sex specific prevalence of overweight and obesity is described in (Table 6).

**Table 6:** Prevalence of overweight /obesity by sex and age groups among preschool children in Gondar City in 2016.

Variables	Overweight/obese (No. (%)	Overweight (No. (%)	Obese (No. (%)
**Overall**	69(13.8)	48(9.6)	21(4.2)
**Sex**			
Male	40(16.5)	22(9.1)	18(7.4)
Female	29(11.3)	26(10.1)	3(1.2)
**Age in months**			
36–47	51(20.4)	36(14.4)	15(6.0)
48–60	18(7.2)	12(4.8)	6(2.4)

Number of total participants, n = 500

### Factors associated with overweight / obesity among preschool children

In this study child age, dietary diversity score, consumption of sweat food and time spent in watching television were positively associated with overweight/obesity while maternal education was negatively associated with overweight/obesity.

Children with age group 36–47 months were 2.38 times more likely to be overweight/obese when compared to children from age group 48–60 months (AOR = 2.38 [95% CI:1.27, 4.46]) (Table 7).

**Table 7:** Factors associated with overweight/obesity among preschool children in Gondar City in 2016.

Variables	Overweight/obese		Crude OR	Adjusted OR
	YES (n = 69) NO (n = 431) No.[%]	No.[%]		
**Age (months)**				
36–47	51(20.4)	199(79.6)	3.30(1.87,5.84)	2.38(1.27,4.46)*
48–60	18(7.2)	232(92.8)	1	1
**Dietary diversity**				
Low	4(7.7)	48(92.3)	1	1
Medium	26(8.2)	290(91.8)	1.07(0.36,3.22)	1.02(0.31,3.39)
High	39(29.5)	93(70.5)	5.03(1.7,14.91)	3.73(1.15,12.54)*
**Sweet foods**				
Yes	60(17.5)	282(82.5)	3.52(1.70,7.29)	2.69(1.21,5.98)*
No	9(5.7)	149(94.3)	1	1
**Mother education**				
No education	9(10.1)	80(89.9)	1	1
Primary	19(13.6)	121(86.4)	1.39(0.6,3.29)	0.87(0.34,2.26)
Secondary	13(7.6)	159(92.4)	0.72(0.29,1.77)	0.35(0.12,0.96)*
Above secondary	28(28.3)	71(71.7)	3.50(1.50,7.92)	1.90(0.74,4.90)
**Time spent in watching TV/playing game**				
≤2hr	26(7.6)	315(92.4)	1	1
>2hr	43(27)	116(73)	4.49(2.64,7.64)	4.01(2.22,7.28)**
**Socio economic status**				
Low	16(9.7)	149(90.3)	1	1
Medium	23(13.7)	145(86.3)	1.47(0.75,2.90)	1.57(0.69,3.58)
High	30(18)	137(82)	2.04(1.07,3.90)	1.93(0.86,4.35)***

Key: P < 0.05*, P <0.001**, p >0.05***

Number of total participants, n = 500

The odds of being overweight/obese for those who had high dietary diversity score was 3.73 times more likely when compared to low dietary diversity score (AOR = 3.73 [95%CI:1.15, 12.54]) (Table 7).

Study participants who consumed sweat foods were 2.69 times more likely to be overweight/obese as compared to those who did not consumed sweat foods (AOR = 2.69 [95%CI:1.21, 5.98]) (Table 7).

Maternal education level was negatively associated with overweight/obesity. Those children whose mothers have secondary education level have 65% less likely chance of being overweight/obese as compared to those who have no formal education. AOR = 0.35 [95%CI; 0.12, 0.96]) (Table 7).

Those preschool children who spent more than 2 hour a day by watching television or playing games had 4.01 times more likely chance of being overweight/obese as compared to those who spent less than 2 hour (AOR = 4.01 [95%CI:2.22, 7.28]) (Table 7).

## Discussion

This study determines the prevalence of overweight/obesity and associated factors among preschool children in Gondar City.

The figures of prevalence of overweight/obesity (13.8% total, 9.6% overweight and 4.2% obese) in this study in Gondar city were comparable to found in Hawassa region of Ethiopia (10.7% total, 7.3% overweight and 3.4% obese) [10]. These findings were also more or less consistent with a study conducted in different countries, in Malawi the combined prevalence was 14.5% with 8.7% overweight and 5.8% obesity [6]. In Mozambique the combined prevalence was 11.9% with 7.7% overweight and 4.2% obesity [6]. In Pernambuco Brazil 2006 the prevalence of overweight was 9.7% [13]. In Basrah,Iraq the combined prevalence was 11.2% with 7.6% overweight and 3.6% obese [14]. This shows many changes are taking place in developing countries, which are of concern. Even if the prevalence of under-nutrition is decreasing in Ethiopia at the same time another public health problem overweight /obesity emerges, it might be due to the change in life style which includes reduced physical activity, adoption of western diets which are high in saturated fat, sugar and refined foods and urbanization has occurred rapidly.

However the present finding was lower than the findings in Iran 35.7% combined prevalence with 12% overweight and 23.7% obesity [15]. In Vietnam 21.1% combined prevalence of overweight and obesity [16], in Brazil 21.9% combined prevalence with 14.4% overweight and 7.5% obese in 2013 [17]. This might be due to socio economic status variation, cultural difference in dietary intake.

The prevalence of this study was also slightly higher than a study conducted in Cameroon, the combined prevalence was 8% with 6.3% overweight and 1.7% obese [18], this might be the difference in the study populations characteristics they included nationwide data while in this study only data from Gondar City was included.

In the present study age was associated with childhood overweight/obesity. The highest prevalence of overweight/obesity observed in the age group of 36–47 months (20.4%) the values being 14.4% for overweight and 6% for obesity. While the lowest prevalence observed in the age group of 48–60 months (7.2%) the values being 4.8% for overweight and 2.4% for obesity. A similar pattern of decreasing prevalence of overweight and obesity with age was reported from Hawassa, Ethiopia and Cameroon [10, 18], this may be during the early childhood period physiologically the percentage of body fat decreases and muscle tissue increases and children get thinner, as well as they have a chance to join school which may attributed to increase physical activity level (55.6%) in age groups from 48–60 months than in physical activity level (44.4%) in age groups from 36–47 months.

The present study showed that a high dietary diversity score had statistically significant association with preschool children overweight/obesity. A higher prevalence of overweight/obesity was observed in those who have higher dietary diversity score (29.5%) than in both medium (8.2%) and low (7.7%) dietary diversity score. Similar finding found in a study conducted in Hawassa, Ethiopia and China [10, 19]. This might be due to diets that offer greater variety foods could increase food intake and energy dense foods might increase body weight. Although children consume several type of food items, the amount of consumption is low for many food groups and vegetables, fruits and legumes are often culturally less desired and possibly mothers tend to give large portion of foods which are energy dense and sweat as children want to eat more.

Consumption of sweet foods in this study was significantly associated with overweight/obesity. Those children who consumed sweet foods were 2.7 times more likely to be overweight/obese as compared to those who didn’t consumed, there were supporting finding from Hawassa, Ethiopia [10] and a systematic review in Netherlands [20]. This can be suggested by sweet foods have a greater likelihood of being accepted by children and are energy dense. If a large portion of diet comes from these foods, it lessens the chance of eating other foods. Using sweet foods as a reward for children may also have effect to consume large amounts and they are also easily available with affordable cost.

Educational status of mothers was negatively associated with overweight/obesity of preschool children. Those preschool children whose mothers had secondary level education had 65% less likely chance of being overweight/obesity as compared to mothers who had no education and this finding is consistent with studies done in Iran and Brazil [13, 15]. This could be due to the fact that more years of schooling for the mothers reflect in higher income and the possibility of acquiring better quality food. Moreover, education provides the capacity to incorporate health recommendations and make healthy choices regarding food products, such as the inclusion of fruits and vegetables in the diet.

This study also shows that preschool children who watch television more than 2 hour per day had 4 times more likely chance of being overweight /obese as compared to those who watch less than 2 hour per day. This result is similar with another study done in Kenya [21],Iraq [14] and Iran [15]. This might be due to reduced physical activity at the time of watching television, high chance of consuming large portion of foods, soft drink and processed food marketing on television encourage children to eat more foods and even may interrupt with sleeping.

## Limitation of study

There was a potential for recall bias in the food frequency questions, also the food frequency questionnaire did not account for portion size.

## Conclusion

In this study the prevalence of overweight/obesity in preschool children was 13.8%. Being in age group of 36–47 month, high dietary diversity score, time spent in watching television greater than 2 hours a day and consumption of sweet foods were positively associated with overweight /obesity, while preschool children whose mothers had secondary education were preventive factors for being overweight/obese. Once considered a high income country problem overweight/obesity is on the rise in Ethiopia particularly in urban cities like Gondar. Therefore this study indicates the need for formulating preventive programs and policies during a child’s early years.

## Ethics approval and consent to participate

Ethical clearance was obtained from Ethical Review Board of University of Gondar, and support letter written by institute of public health was given to Gondar City Health Bureau and to each selected sub city administration.

A support letter from local authorities and concerned government bodies was obtained, after getting permission from selected sub-cities to participate in the study, an informed verbal consent was obtained from mothers or caretaker’s. Those who are not willing to take part in the study were excluded.

The data obtained from them were kept confidential by not writing participant`s name in the questionnaire and during interview.

## Supporting information

S1 Data This is the available data. (SAV)

## Abbreviations

AOR: Adjusted Odds Ratio
BMI: Body Mass Index
CDC: Center for Disease Prevention and Control
CI: Confidence Interval
CSA: Central Statistics Agency
KG: Kindergarten
OR: Odds Ratio
SES: Socio Economic Status
SPSS: Statistical Package for Social Science
SSA: Sub-Saharan Africal
TV: Television
USA: United States of America
WHO: World Health Organization
